# Statistics of the Medical Colleges of the U. S.

**Published:** 1840

**Authors:** 


### BIBLIOGRAPHICAL NOTICES.

---

Art. XVII.—*Statistics of the Medical Colleges of the United States.* By T. Romeyn Beck, M. D. (Extracted from the Transactions of the Medical Society of the State of New York, vol. iv., 1839.

Professor Beck, with that industry in the collection and collocation of statistical *data* for which he is so distinguished, has brought together in the work before us, a numerous and interesting body of facts, relative to our medical schools. If some of them are not altogether correct, which we believe to be the case, the fault does not lie in the author but the nature of his task. Scattered over and owing allegiance to so many states of the union, fluctuating in their condition, too often prone to exaggeration in their reports, and irregular in the publication of them, our Medical Institutions, more, perhaps, than any others in the Union, set the historian at defiance ; and we are more surprised at Dr. Beck's general success in this undertaking, than at the omissions which it exhibits.

For the purpose of ascertaining the number of students in attendance on all the schools of the different States, at a period as near as possible to the present, we have constructed from Prof. Beck's work, supplying two or three omissions, the following table for 1838–'9 :

| | | | |
|---|---|---|---|
| University of Pennsylvania, | 402 | Students. | |
| Jefferson Medical College, | 230 | " | |
| Transylvania University, | 211 | " | |
| Medical College of the State of South Carolina | 151 | " | |
| Louisville Medical Institute, Ky., | 120 | " | |
| Castleton Medical School, Vermont, | 115 | " | |
| College of physicians, and surgeons of the Western District of New York, Fairfield, | 114 | " | |
| Cincinnati College, | 114 | " | |
| Medical College of Ohio, | 101 | " | |
| College of physicians and surgeons of the State of New York, | 96 | " | |
| Harvard University, | 85 | " | |
| Berkshire Medical Institution, Ms., | 85 | " | |
| Dartmouth College, | 78 | " | |
| Bowdoin College, Maine, | 77 | " | |
| Geneva College, New York, | 76 | " | |
| Albany Medical College, New York, | 68 | " | |
| Woodstock Medical School, Vermont, | 65 | " | |
| University of Virginia, | 60 | " | |
| Medical College of Georgia, | 60 | " | |
| Washington Medical College, Baltimore, | 40 | " | conjectural. |
| Medical Department of Hampden Sidney College, Richmond, Va., | 48 | " | |
| Yale College, | 46 | " | |
| Willoughby University, Ohio, | 40 | " | |
| Medical College, South Carolina, | 29 | " | |
| Medical College of Louisiana, | 25 | " | " |
| Total: | 2536 | | |

Thus it will be perceived, that the twenty-five schools in operation in the winter of 1838–'9 had an average of 101.44 each—for the sake of round numbers, we shall say one hundred. Of the whole number of schools, eight were above this average, and seventeen below it. Six of the schools or less than one-third of the whole, had half the entire number of pupils, and the University of Pennsylvania alone, had one-sixth of the aggregate.

Taking the great regions of the Union separately, we find that the whole number of students in the eleven schools of

New England and New York was nine hundred and five—,in the five schools of the middle States, seven hundred and eighty,—in the four schools of the South, two hundred and sixty-five,—and in the five schools of the West, five hundred and eighty-six. Thus the eastern institutions fall, respectively, on an average, 17. 75 per cent. below the general average; the middle rise fifty-six above it; the southern fall 33. 75 per cent. below, and the western rise 17. 20 per cent. above. In the eastern division two elevenths only of the schools or eighteen per cent. of the group rise above the average; in the middle two fifths or forty per cent.; in the southern one fourth or twenty-five per cent., and in the western four-fifths or eighty per cent. Thus it results, that the proportional number of schools in the different regions, into which we have divided the whole country, which rise, in the number of their students, above the average of the whole, is as follows:—Western eighty per cent., Middle forty, Southern twenty-five, Eastern eighteen.

The rank of the different States which have established schools, will appear from the following table constructed from the *data* furnished by Dr. Beck:

| | | | |
|---|---|---|---|
| 1. Pennsylvania in - - - | 2 | schools, | 632 |
| 2. New York in - - - - - | 4 | do | 354 |
| 3. Kentucky in - - - - - | 2 | do | 331 |
| 4. Ohio in - - - - - - - | 3 | do | 255 |
| 5. South Carolina in - - - | 2 | do | 180 |
| 6. Vermont in - - - - - - - | 2 | do | 180 |
| 7. Massachusetts in - - - - | 2 | do | 170 |
| 8. Virginia in - - - - - - | 2 | do | 108 |
| 9. New Hampshire in - - - | 1 | do | 78 |
| 10. Maine in - - - - - - - | 1 | do | 77 |
| 11. Georgia in - - - - - - | 1 | do | 60 |
| 12. Connecticut in - - - - | 1 | do | 46 |
| 13. Maryland in - - - - - - | 1 | do | 40 |
| Louisiana in - - - - - - - | 1 | do | 25 |
| | | | 2536 |

Such was the order of the States in 1838–'9, but in 1839-'40, Kentucky had four hundred and sixty pupils, and was ahead of New York—in other words, was the second State in the Union, in the number of medical students in attendance on lectures in her colleges. Fourteen of the States have established medical schools; thirteen others, with two territories fast advancing to the dignity of States, remain to authorize similar institutions, and it may be confidently predicted, that many of them will do so—for this there is no *prophylactic.*

Let us turn our attention, for a moment, to the annual income which these establishments yield to their professors. Our estimate can be but an approximation to the truth as many *data* are wanting. In every medical school there are four classes of matriculated pupils, who do not pay. 1st. resident graduates. 2d. Students who had paid previously for two courses of lectures. 3d. Students who get their tickets on credit and never pay. 4th. Charity pupils. If we estimate each of the two former groups at five per cent. of the whole, and the two latter combined at ten per cent. they will amount to five hundred and seven; but as we believe some of the estimates too low, we shall assume five hundred and thirty-six, which deducted from twenty-five hundred and thirty-six, the whole number of pupils in the schools of the Union, leaves two thousand who pay. In many of our institutions, the ticket of each Professor is eight, ten, or twelve dollars, in some of them twenty, in the majority fifteen, we shall take fifteen as the average; some of the schools have eight Professorships, a greater number seven, a few only five; we shall not be far from the truth to average them at six; which multiplied by fifteen, gives ninety dollars, as the sum which each pupil pays to the professors of the school which he attends. Now if we multiply two thousand by ninety we have one hundred and eighty thousand dollars as the aggregate of professional fees; which divided among one hundred and fifty, gives a quotient of twelve hundred dollars for each. The matriculation and graduation fees may be placed against the contingent expenses.

If the price of the ticket and the number of pupils, were the same in all our schools, the same sum, twelve hundred dollars, would be received by each Professor; but as more than one fourth of the pupils attend schools in which the price of the ticket is twenty dollars, it follows that the professors of those schools receive more than the average, while others receive less—when the number of students is the same. But there are great inequalities in the relative number of students in different schools, and, therefore the yearly income of many professors must fall far below twelve hundred dollars; not a few indeed, receive but five, four, or even three hundred dollars.

If we estimate all other expenditures of a student from the time he leaves home till he returns, as equal to what he pays for tuition, we have an aggregate of three hundred and sixty thousand dollars, from two thousand pupils; but to this, must be added, the sum expended, otherwise than for tickets, by five hundred and thirty-six students who do not pay for tuition, which gives a grand total of four hundred and fifty thousand four hundred and eighty dollars, expended annually, on the collegiate instruction of those who are to fill up the ranks of the profession. Such an appropriation ought to supply the country with an adequate number of well educated physicians and surgeons, but this seems not to be the fact, for we every where find practitioners, who have not attended lectures in any school.

Professor Beck has furnished *data* for other estimates, which would not be without interest, but we shall leave it with our readers to supply our omissions. D.

